# The positive impact of smoking cessation on fracture risk in a nationwide cohort study

**DOI:** 10.1038/s41598-024-60301-5

**Published:** 2024-04-30

**Authors:** Jin-Sung Park, Kyung-Chung Kang, Se-Jun Park, Jeong-Keun Kim, Kyungdo Han, Jae-Young Hong

**Affiliations:** 1 Department of Orthopedics, Samsung Medical Center, Sungkyunkwan University School of Medicine, 81 Irwon-ro, Gangnam-gu, Seoul, 06351 South Korea; 2 Department of Orthopedics, Kyung Hee University Hospital, Kyung Hee University School of Medicine, 23 Kyungheedaero, Dongdaemun‑gu, Seoul, 02447 South Korea; 3 Department of Statistics and Actuarial Science, Soongsil University, 369 Sangdo-ro, Dongjak-gu, Seoul, 06978 South Korea; 4 Department of Orthopedics, Korea University Ansan Hospital, 123 Jeokgeum-ro, Danwon gu, Ansan, Gyeonggi 15355 South Korea

**Keywords:** Health care, Medical research

## Abstract

Many studies sought to demonstrate the association between smoking and fracture risk. However, the correlation between smoking and fractures remains controversial. This study aimed to examine the impact of smoking and smoking cessation on the occurrence of fractures using prospective nationwide cohort data. We enrolled those who underwent a National Health Insurance Service (NHIS) health checkup in 2009–2010 who had a previous health checkup 4-year prior (2005–2006). The study population of 4,028,559 subjects was classified into three groups (non-smoker, smoking cessation, current smoker). The study population was also analyzed according to fracture type (all fractures, vertebral fracture, hip fracture). Lastly, the smoking cessation group and current smoker group were divided into four subgroups based on a lifetime smoking amount cut-off of 20 pack-years (PY). Multivariate-adjusted hazard ratios (HRs) of fracture were examined through a Cox proportional hazards model. After multivariable adjustment, non-smokers showed the lowest risk of fracture (HR = 0.818, CI 0.807–0.828, p < 0.0001) and smoking cessation significantly lowered the risk of fracture (HR 0.938, 95% CI 0.917–0.959, p < 0.0001) compared to current smokers. Regardless of 20PY, all smoking cessation subgroups showed significantly less risk of fractures than current smokers with ≥ 20PYs. Smoking increases the risk of fracture, and smoking cessation lowers the risk of fracture.

## Introduction

With an estimated 42.1 million cigarette smokers in the United States, leading to 480,000 death annually, smoking has always been a major public health concern^1^. To reduce the prevalence of smoking, regulations were enacted that restrict advertising, reinforce warnings on cigarette packages, and increase smoke-free areas. However, in South Korea, the prevalence of cigarette smoking among male and female adults is still high^2,3^.

Smoking is a major lifestyle factor that is related to osteoporosis, and several studies have already proven that smoking is associated with low bone mineral density (BMD)^4–6^. However, low BMD alone does not account for fractures; a variety of other factors are involved in fracture risk. Thus, many studies tried to clarify the association between smoking and fracture risk^7–15^. Some studies demonstrated no relationship between smoking and factures^16,17^. Such inconsistent research findings could be the result of subject differences or study-specific variable adjustments.

The best way to demonstrate how smoking affects fractures is to identify the actual fracture incidence in a generally representative population, since it is hard to account for all fracture-related risk variables. Additionally, if the incidence of fracture changes after smoking cessation, this might be a valid method for demonstrating the negative impacts of smoking.

Therefore, the aim of this study was to examine the impact of smoking and smoking cessation on the occurrence of fractures using prospective cohort data from a national data set (Korean Health Insurance Review and Assessment, HIRA).

## Results

### Baseline characteristics

Of the 4,028,559 subjects, 75.51% (n = 3,042,141) never smoked, 4.79% quit smoking (n = 192,784) and 19.70% continued to smoke (n = 793,634). Overall, smoking rates were higher in males than females and males made up the majority of smoking cessation and current smoker groups. The mean age was 56.2 ± 10.04, 53.09 ± 9.52, and 51.94 ± 9.34 years in non-smoker, smoking cessation, and current smoker groups, respectively. The amount of smoking in past smokers was 21.9 pack-years (PY) and current smokers was 22.4 PY. The mean follow-up period was 6.53 ± 1.35 years. The distribution of demographic variables and chronic disease is shown in Table 1.

**Table 1:** Baseline characteristics of the study populations according to smoking status.

	Non-smoker	Smoking cessation	Current smoker
N (%)	3,042,141 (75.51)	192,784 (4.79)	793,634 (19.70)
Sex			
Male, N (%)	773,894 (25.44)	188,069 (97.55)	769,806 (97)
Female, N (%)	2,268,247 (74.56)	4715 (2.45)	23,828 (3)
Age, mean (SD)	56.2 ± 10.04	53.09 ± 9.52	51.94 ± 9.34
≥ 65, N (%)	2,375,123 (78.07)	165,712 (85.96)	700,427 (88.26)
Amount of smoking, pack-years, mean (SD)	0	21.92 ± 16.57	22.42 ± 13.94
Alcohol drinking, N (%)	57,572 (1.89)	27,107 (14.06)	138,638 (17.47)
Low income, N (%)	586,968 (19.29)	25,359 (13.15)	116,707 (14.71)
Regular exercise, N (%)	636,853 (20.93)	52,356 (27.16)	154,781 (19.5)
Body mass index ≥ 25, N (%)	2,059,369 (67.69)	112,180 (58.19)	527,600 (66.48)
Abdominal obesity, N (%)	1,103,307 (36.27)	52,203 (27.08)	174,727 (22.02)
Diabetes, N (%)	311,983 (10.26)	27,527 (14.28)	103,716 (13.07)
Hypertension, N (%)	1,031,243 (33.9)	68,965 (35.77)	239,910 (30.23)
Hyperlipidemia, N (%)	775,495 (25.49)	50,349 (26.12)	166,083 (20.93)
Chronic kidney disease, N (%)	256,152 (8.42)	14,504 (7.52)	46,627 (5.88)
Prevalence of chronic obstructive pulmonary disease, N (%)	223,901 (7.36)	13,574 (7.04)	48,871 (6.16)
Prevalence of ischemic heart disease, N (%)	200,829 (6.6)	15,464 (8.02)	37,094 (4.67)
Prevalence of ischemic stroke, N (%)	76,203 (2.5)	5501 (2.85)	12,239 (1.54)
Prevalence of chronic heart failure, N (%)	31,237 (1.03)	1603 (0.83)	4051 (0.51)
Prevalence of end stage renal disease, N (%)	1879 (0.06)	149 (0.08)	192 (0.02)
Prevalence of cancer, N (%)	83,410 (2.74)	6845 (3.55)	9232 (1.16)
Osteoporosis, N (%)	366,944 (12.06)	3175 (1.65)	10,474 (1.32)
Rheumatoid arthritis, N (%)	4736 (0.16)	101 (0.05)	326 (0.04)
Steroid use, N (%)	69,855 (2.3)	4948 (2.57)	15,914 (2.01)
Event, N (%)			
All fracture, N (%)	299,303 (9.84)	9704 (5.03)	40,801 (5.14)
Vertebral fracture, N (%)	120,865 (3.97)	3730 (1.93)	15,637 (1.97)
Hip fracture, N (%)	14,329 (0.47)	672 (0.35)	2901 (0.37)
F/U duration			
Years, mean (SD)	6.5 ± 1.39	6.63 ± 1.22	6.64 ± 1.22
Median (Q1–Q3)	6.75 (6.22–7.32)	6.85 (6.25–7.35)	6.97 (6.26–7.34)

All characteristics met p < 0.0001.

Alcohol drinking indicates drinking more than 30 g of alcohol per day.

Low income indicates income below the 20th percentile.

Regular exercise indicates moderate exercise more than 3 days per week or vigorous exercise more than 3 days per week.

Abdominal obesity indicates wait circumference ≥ 90 cm in male and ≥ 85 cm in female.

Osteoporosis indicates Mean T-score of lumbar spine or femur ≤ − 2.5

Steroid use indicates a history of daily intake of more than 5 mg of steroids.

### Effects of smoking on fracture outcomes

According to the demographic data, all fracture incidence was higher in the non-smoker group (9.84%) than in the current smoker group (5.14%), while the smoking cessation group (5.03%) showed the lowest fracture incidence (Table 1).

When separated by sex, fracture incidence was similar between the smoking cessation (4.86%) and current smoker (4.87%) groups among males, but in females, the incidence of fracture was lower in the smoking cessation (11.92%) group than in current smokers (13.85%). The actual incidence of fracture was lower in the smoking cessation group (incidence rates (IR) 0.98 per 1000 person-years) than in non-smokers and current smokers (Table 2).

**Table 2:** Comparison of fracture incidence and follow up duration according to smoking and gender.

	Male				Female			
	Non-smoker	Smoking Cessation	Current Smoker	p	Non-smoker	Smoking Cessation	Current Smoker	p
N	773,894	188,069	769,806		2,268,247	4715	23,828	
Age, mean (SD)	56.19 ± 10.91	53.03 ± 9.51	51.79 ± 9.28	< .0001	56.2 ± 9.72	55.46 ± 9.48	56.65 ± 10.25	< .0001
≥ 65, N (%)	190,994 (24.68)	26,246 (13.96)	87,804 (11.41)	< .0001	476,024 (20.99)	826 (17.52)	5403 (22.68)	< .0001
Amount of smoking, pack-years, Mean (SD)		22.25 ± 16.57	22.76 ± 13.89	< .0001		8.82 ± 10.16	11.25 ± 10.52	< .0001
Event, N (%)								
All fracture, N (%)	41,160 (5.32)	9142 (4.86)	37,502 (4.87)	< .0001	258,143 (11.38)	562 (11.92)	3299 (13.85)	< .0001
Vertebral fracture, N (%)	17,653 (2.28)	3508 (1.87)	14,168 (1.84)	< .0001	103,212 (4.55)	222 (4.71)	1469 (6.17)	< .0001
Hip fracture, N (%)	3192 (0.41)	642 (0.34)	2706 (0.35)	< .0001	11,137 (0.49)	30 (0.64)	195 (0.82)	< .0001
**F/U duration**								
Years, mean (SD)	6.63 ± 1.23	6.64 ± 1.21	6.65 ± 1.2	< .0001	6.46 ± 1.44	6.37 ± 1.51	6.34 ± 1.58	< .0001
Median (Q1–Q3)	6.83 (6.28–7.36)	6.86 (6.26–7.35)	7.01 (6.26–7.34)	< .0001	6.72 (6.21–7.31)	6.68 (6.15–7.25)	6.71 (6.15–7.28)	0.0003

After multivariable adjustment, non-smokers showed the lowest risk of fracture (Model 5e; hazard ratio (HR) 0.82, 95% confidence interval (CI) 0.81–0.83, p < 0.0001) and smoking cessation significantly lowered the risk of fracture (Model 5e; HR 0.94, 95% CI 0.92–0.96, p < 0.0001) compared to current smokers. In the analysis of vertebral fracture and hip fracture, smoking cessation consistently lowered risk compared to current smokers (Model 5e; HR 0.92, 95% CI 0.89–0.95; HR 0.89, 95% CI 0.82–0.97, respectively, p < 0.0001) (Table 3).

**Table 3:** Incidence rate (IR) and multivariate-adjusted hazard ratios (HRs) (95% confidence intervals) of fractures.

Smoking status	N	Events	IR	HR (95% CI)				
				Model 1a	Model 2b	Model 3c	Model 4d	Model 5e
All fractures								
Non-smoker	3,042,141	299,303	15.13	1.95 (1.93, 1.97)	0.79 (0.78, 0.80)	0.81 (0.80, 0.82)	0.81 (0.80, 0.82)	0.82 (0.81, 0.83)
Smoking cessation	192,784	9704	7.59	0.98 (0.959, 1.002)	0.92 (0.90, 0.94)	0.939 (0.92, 0.96)	0.94 (0.92, 0.96)	**0.94 (0.92**, **0.96)**
Current smoker	793,634	40,801	7.75	1 (Ref.)	1 (Ref.)	1 (Ref.)	1 (Ref.)	1 (Ref.)
Vertebral fracture								
Non-smoker	3,042,141	120,865	6.11	2.06 (2.03, 2.09)	0.74 (0.72, 0.75)	0.76 (0.75, 0.78)	0.76 (0.75, 0.78)	0.77 (0.75, 0.78)
Smoking cessation	192,784	3730	2.92	0.98 (0.95, 1.02)	0.90 (0.87, 0.94)	0.92 (0.89, 0.95)	0.92 (0.89, 0.96)	**0.92 (0.89**, **0.95)**
Current smoker	793,634	15,637	2.97	1 (Ref.)	1 (Ref.)	1 (Ref.)	1 (Ref.)	1 (Ref.)
Hip fracture								
Non-smoker	3,042,141	14,329	0.72	1.32 (1.27, 1.37)	0.60 (0.57, 0.63)	0.65 (0.62, 0.68)	0.65 (0.62, 0.68)	0.65 (0.62, 0.69)
Smoking cessation	192,784	672	0.53	0.96 (0.88, 1.04)	0.85 (0.78, 0.93)	0.90 (0.83, 0.98)	0.90 (0.82, 0.98)	**0.89 (0.82**, **0.97)**
Current smoker	793,634	2901	0.55	1 (Ref.)	1 (Ref.)	1 (Ref.)	1 (Ref.)	1 (Ref.)

IR (Incidence rate) indicates per 1000 person years.

Significant values are given in bold.

^a^Non-adjusted.

^b^Adjusted for age and sex.

^c^Adjusted for age, sex, alcohol drinking, low income, regular exercise and body mass index.

^d^Adjusted for age, sex, alcohol drinking, low income, regular exercise, body mass index, diabetes, hypertension and hyperlipidemia.

^e^Adjusted for age, sex, alcohol drinking, low income, regular exercise, body mass index, diabetes, hypertension, hyperlipidemia, chronic kidney disease, chronic obstructive pulmonary disease, ischemic heart disease, stroke, chronic heart failure, steroid use, osteoporosis and rheumatoid arthritis.

### Fracture outcomes according to smoking PY

After multivariable adjustment, regardless of 20 PY, all smoking cessation subgroups showed significantly lower risk of fractures, including vertebral and hip fractures, than current smokers with ≥ 20 PY in the fully adjusted model. Smoking cessation with < 20 PY was associated with lower risk of fractures than smoking cessation with ≥ 20 PY in all fracture and vertebral fracture groups. In contrast, smoking cessation with < 20 PY led to higher risk of hip fracture compared with smoking cessation with ≥ 20 PY (HR 0.95, 95% CI 0.83–1.09 vs. HR 0.88, 95% CI 0.80–0.98, p < 0.0001). Also, current smokers with < 20 PY showed higher risk for hip fracture than current smokers with ≥ 20 PY (HR 1.06, 95% CI 0.98–1.14 vs. 1 (Ref.), p < 0.0001) (Table 4).

**Table 4:** Incidence rate (IR) and multivariate-adjusted hazard ratios (HRs) (95% confidence intervals) of fractures according to pack-year.

Smoking status	N	Events	IR	HR (95% CI)				
				Model 1a	Model 2b	Model 3c	Model 4d	Model 5e
All fractures								
Non-smoker	3,042,141	299,303	15.13	1.80 (1.78, 1.83)	0.77 (0.76, 0.79)	0.81 (0.80, 0.82)	0.81 (0.80, 0.82)	0.81 (0.80, 0.83)
Smoking cessation/PY < 20	91,548	3836	6.26	0.75 (0.72, 0.77)	0.88 (0.85, 0.91)	0.91 (0.89, 0.94)	0.91 (0.88, 0.94)	**0.91 (0.88**, **0.94)**
Smoking cessation/PY ≥ 20	101,236	5868	8.82	1.05 (1.02, 1.08)	0.94 (0.91, 0.96)	0.95 (0.93, 0.98)	0.95 (0.93, 0.98)	**0.95 (0.93**, **0.98)**
Continuous smoker/PY < 20	332,720	15,235	6.86	0.82 (0.80, 0.83)	0.96 (0.95, 0.98)	0.99 (0.97, 1.01)	0.99 (0.93, 1.01)	0.99 (0.97, 1.01)
Continuous smoker/PY ≥ 20	460,914	25,566	8.39	1 (Ref.)	1 (Ref.)	1 (Ref.)	1 (Ref.)	1 (Ref.)
Vertebral fracture								
Non-smoker	3,042,141	120,865	6.11	1.81 (1.77, 1.84)	0.71 (0.70, 0.73)	0.75 (0.73, 0.76)	0.74 (0.73, 0.76)	0.75 (0.73, 0.77)
Smoking cessation/PY < 20	91,548	1304	2.13	0.63 (0.59, 0.67)	0.82 (0.77, 0.86)	0.85 (0.80, 0.90)	0.85 (0.80, 0.90)	**0.85 (0.80**, **0.90)**
Smoking cessation/PY ≥ 20	101,236	2426	3.65	1.08 (1.03, 1.13)	0.91 (0.87, 0.95)	0.93 (0.89, 0.97)	0.93 (0.89, 0.97)	**0.93 (0.89**, **0.97)**
Continuous smoker/PY < 20	332,720	5320	2.40	0.71 (0.68, 0.73)	0.91 (0.88, 0.94)	0.94 (0.91, 0.97)	0.94 (0.91, 0.97)	0.94 (0.91, 0.97)
Continuous smoker/PY ≥ 20	460,914	10,317	3.39	1 (Ref.)	1 (Ref.)	1 (Ref.)	1 (Ref.)	1 (Ref.)
Hip fracture								
Non-smoker	14,329	14,329	0.72	1.16 (1.11, 1.22)	0.61 (0.57, 0.64)	0.66 (0.62, 0.70)	0.66 (0.63, 0.70)	0.67 (0.64, 0.71)
Smoking cessation/PY < 20	237	237	0.39	0.62 (0.54, 0.71)	0.89 (0.78, 1.02)	0.96 (0.84, 1.10)	0.96 (0.84, 1.10)	**0.95 (0.83**, **1.09)**
Smoking cessation/PY ≥ 20	435	435	0.65	1.05 (0.94, 1.16)	0.84 (0.76, 0.94)	0.90 (0.81, 1.00)	0.89 (0.80, 0.99)	**0.88 (0.80**, **0.98)**
Continuous smoker/PY < 20	994	994	0.45	0.72 (0.66, 0.77)	1.03 (0.96, 1.11)	1.06 (0.98, 1.14)	1.06 (0.99, 1.15)	1.06 (0.98, 1.14)
Continuous smoker/PY ≥ 20	1907	1907	0.63	1 (Ref.)	1 (Ref.)	1 (Ref.)	1 (Ref.)	1 (Ref.)

IR (incidence rate) indicates per 1000 person years.

*PY* pack-year.

Significant values are given in bold.

^a^Non-adjusted.

^b^Adjusted for age and sex.

^c^Adjusted for age, sex, alcohol drinking, low income, regular exercise and body mass index.

^d^Adjusted for age, sex, alcohol drinking, low income, regular exercise, body mass index, diabetes, hypertension and hyperlipidemia.

^e^Adjusted for age, sex, alcohol drinking, low income, regular exercise, body mass index, diabetes, hypertension, hyperlipidemia, chronic kidney disease, chronic obstructive pulmonary disease, ischemic heart disease, stroke, chronic heart failure, steroid use, osteoporosis and rheumatoid arthritis.

## Discussion

This is a prospective cohort study that examined the effects of smoking on fractures based on nationwide administrative data. In this study, the risk of fractures including vertebral and hip fracture was the lowest in the non-smoker group in a fully adjusted model. Smoking cessation with < 20 PY significantly lowered risk of fracture including vertebral fracture compared to the current smoker group. The major finding of this study was that smoking cessation lowered the risk of fracture compared to those who continued to smoke.

Smoking is associated with lower body mass^18^, earlier onset of menopause^19^, aggravation of oxidative stress^20^, and a more unhealthy and sedentary lifestyle. Also, smoking has a detrimental effect on bone metabolism in several studies^21–23^. Several mechanisms might explain the increased risk of fracture among smokers: 1. Direct effects on bone cells, 2. Reductions in blood flow to bone, 3. Lower calcium absorption, 4. Changes in vitamin D metabolism to lower vitamin D in the blood, or 5. Increased catabolism of estrogen. In addition, smoking is associated with BMD^4–6^. The effect of smoking on BMD and fractures may result from changes in serum estradiol^24^, serum parathyroid^25^, and serum vitamin D^26^ levels, consequently affecting gastrointestinal calcium absorption and bone cell proliferation^27^, and thus exerting an inhibitory effect on BMD and bone metabolism.

In this study, the crude incidence rate of fractures was higher in the non-smoker group than the smoking cessation and current smoker groups. However, the mean age of the non-smoker group was significantly higher than other groups. Also, the crude incidence rate of fractures in females was higher than in males. According to a previous study, the incidence of osteoporosis in Korean women is about 39%^28^. Estrogen is a factor that affects bone quality, and decreases rapidly with age, especially after menopause. Thus, this result may be the consequence of the effects of age, menopause, and osteoporosis. After adjusting for age and sex, the non-smoker group showed the lowest fracture risk compared to smoking cessation and current smoker groups.

According to a meta-analysis conducted by Vestergaard et al.^29^, in comparison to non-smokers, smokers had a 1.39-fold higher incidence of hip fracture and a 1.76-fold higher incidence of vertebral fracture. In addition, the incidence rate of fracture regardless of site was 1.26 times higher. In smokers, the actual fracture risk rate is higher than the risk rate evaluated only based on BMD, because the change in microstructure of medullary bone caused by smoking weakens resistance to physical force regardless of BMD^30^. In this study, we consistently identified that non-smokers showed the lowest risk of fractures compared to the smoking cessation and current smoker groups in a fully adjusted model. Furthermore, smoking cessation had a positive impact on fracture risk. This is thought to be the most accurate representation of the effect of smoking on fracture occurrence.

Ampelas et al.^31^ reviewed studies on the correlation between smoking and hip fracture. The incidence of hip fracture was found to be higher in current smokers. Former smokers had a slightly increased risk of hip fracture compared to non-smokers, and this risk was inversely correlated with cessation span. Former smokers achieved reduced risk after cessation, but the benefit was not observed until 10 years after cessation. There was not any difference in fracture risk between current smokers and former smokers with less than 5 years cessation of smoking. Cessation ≥ 5 years was associated with a small decrease in the risk of hip fracture and cessation for > 10 years had a benefit on fracture risk.

Our study showed that there was a benefit of lower smoking amount (calculated by PY) in all fractures and vertebral fracture. However, despite the smoking cessation group consistently showed lower risk of fracture compared to the current smoker group regardless of PY, lower smoking amount did not reduce the risk of hip fracture. In this study, especially in hip fracture, the association with the amount of smoking for risk of fracture is thought to be less than that of other fractures such as vertebral fracture. We hypothesize that there are hidden mechanisms or confounding factors that cause the difference between hip and vertebral fracture.

The noteworthy strength of this study is its ability to establish a connection between smoking cessation and fracture risk in a real-world population, utilizing a comprehensive national dataset. However, certain limitations need to be acknowledged. First, given that only participants with access to their medical examination records and a four-year smoking history were included, the potential for selection bias cannot be discounted in this study. Second, this study arises from its 'point prevalence' design, which restricts the analysis to a singular time point for smoking cessation, hindering our ability to determine the specific starting moment and duration of smoking discontinuation. Consequently, any changes in smoking habits post-health examination may introduce errors in result interpretation. Third, although many factors that could be considered risk factors for fractures were adjusted for through multivariable models, we still could not rule out the possibility that other unknown factors may have biased this analysis. Finally, because the results of the current analysis were mainly based on data from a South Korean population, these findings may not be generalizable to other populations.

## Conclusion

This study demonstrated that smoking increases risk of fracture, and that smoking cessation lowers risk of fracture in a nationwide dataset using a multivariate-adjusted model. However, unlike risk of all fracture and vertebral fracture, a lower amount of lifetime smoking did not reduce the risk of hip fracture. More in-depth research is needed to back up these findings.

## Methods

### Data source

The National Health Insurance Service (NHIS) provides health insurance to more than 97% of Koreans. Every one or two years, the NHIS offers routine health checkups to almost all Korean individuals over the age of 40. Health checkup data and national insurance claims records are organized by the NHIS to provide extensive data for medical research, including patient demographics and hospital records. This information includes examinations, laboratory findings, diagnoses (10th revised codes of the International Classification of Diseases, ICD-10), and treatments.

### Study cohort establishment

**Figure 1:**
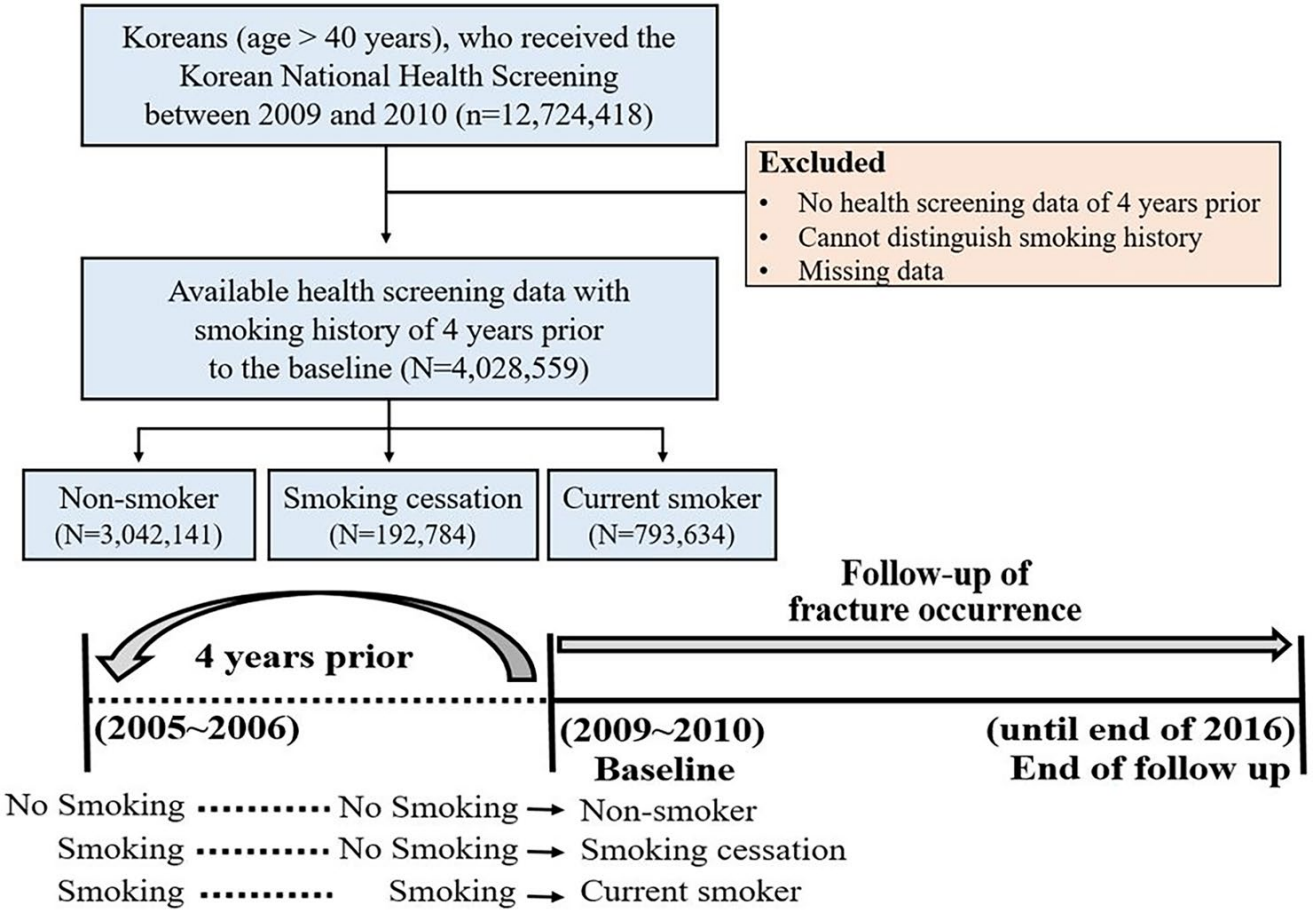
The flow chart of study population.

NHIS health checkup participants from 2009 and 2010 who also underwent a health checkup 4 years prior (2005, 2006) were included in this study. A total of 12,724,418 Korean adults over 40 years of age participated in national health screenings between 2009 and 2010. Of these, 4,028,559 people who did not have missing data and whose health screening data including smoking history from 4 years prior could be accessed. Fracture occurrence was followed from the time of health examinations in 2009 and 2010 until December 31, 2016. Smoking history was determined by self-reported questionnaires including current smoking status, duration, and amount of smoking. Regarding their current smoking status, the participants were given three options: never smoked, smoked in the past but not currently, or continuing to smoke. The study population was classified into three groups (non-smoker, smoking cessation, current smoker). The non-smoker group included those who had consistently not smoked at the health checkup before and after 4 years, and the smoking cessation group included subjects who had smoked before 4 years but had quit smoking at the checkup after 4 years. Those who described smoking at both checkups were considered to be current smokers (Fig. 1).

### Data collection of diagnosed fractures

We reviewed all participant international classification of disease (ICD) code claims until December 31, 2016. The occurrence of vertebral fracture was defined as ICD-10 codes S22.0, S22.1, S32.0, M48.4 and M48.5 claimed at least twice with outpatients. The occurrence of hip fracture was defined as ICD-10 codes S72.0 and S72.1 claimed more than once with hospitalization. All fractures were defined to include other fractures, including upper arm fractures (S42.0, S42.2, and S42.3), forearm fractures (S52.5 and S52.6), and lower leg fractures (S82.3, S82.5, and S82.6) as well as vertebral and hip fractures and claimed at least twice with outpatients.

### Data collection of baseline characteristics

Male and female Korean adults over the age of 40 were included in this study. Through the use of a standardized questionnaire, socio-behavioral data including drinking, regular physical activity, and income were gathered. Body mass index (BMI) and abdominal obesity were assessed in the usual manner^32,33^. ICD-10 codes from prior claims records were used to assess hypertension (ICD-10, I10-15), diabetes (ICD-10, E11-14), hyperlipidemia (ICD-10, E78), chronic kidney disease (ICD-10, N18-19), chronic obstructive pulmonary disease (ICD-10, J41-44), ischemic heart disease (ICD-10, I21-22), ischemic stroke (ICD-10, I63-64), chronic heart failure (ICD-10, I50), end-stage renal disease (ICD-10, N18-19 with renal replacement therapy), cancer (ICD-10, beginning with “C”), and rheumatoid arthritis (ICD-10, M05-06). Osteoporosis was defined as a mean T-score of the lumbar spine or femur ≤ -2.5. Steroid use was defined as a history of daily intake of more than 5 mg of steroids.

### Statistical analysis

Variance analysis was used to analyze continuous variables and chi-squared test was used to analyze categorical variables. A Cox proportional hazards model with a 95% CI was used to analyze HRs. First, the incidence of fractures according to smoking status was analyzed separately in males and females. Second, we conducted overall analysis by calculating the crude incidence rate (IR) and the hazard ratios (HR) of fractures. With the current smoker group serving as a reference, HRs were compared between the smoking cessation, non-smoker, and current smoker groups. We conducted multivariate adjustment using confounders from a non-adjusted model to a fully adjusted model to examine HR. Third, to demonstrate the effects of smoking cessation on outcomes, the smoking cessation group and current smoker group were divided into four subgroups based on 20 PY, and with continuous smokers with ≥ 20 PY serving as a reference, and the HRs of each divided subgroup were compared with non-smokers. Statistical analyses were performed using SAS version 9.3 (SAS Institute Inc., Cary, NC, USA).

### Ethical statement

This study was approved by the Institutional Review Board (IRB) of Korea University Ansan Hospital (approval number: 2019AS0026), which issued a waiver regarding the need for informed consent. All methods were performed in accordance with relevant guidelines and regulations.

## Data Availability

The datasets generated during and/or analysed during the current study are available from the corresponding author on reasonable request.
